# Laparoscopic Diagnosis and Treatment of Gossypiboma Postconventional Ovariohysterectomy in a Bitch

**DOI:** 10.1155/2021/5381079

**Published:** 2021-08-19

**Authors:** Maurício Veloso Brun, Paula Cristina Basso, Marília Teresa de Oliveira, Fabíola Dalmolin, Saulo Tadeu Lemos Pinto-Filho, Helen Fialho Hartmann, Stephanie Lanzarini Abati, Marco Augusto Machado-Silva, Daniel Curvello de Mendonça Müller, Francisco Miguel Sanchez-Margallo

**Affiliations:** ^1^Universidade Federal de Santa Maria (UFSM), Santa Maria-RS, Brazil; ^2^Hospital Veterinário Universitário-UFSM, Santa Maria-RS, Brazil; ^3^Universidade Federal do Pampa (UNIPAMPA), Uruguaiana-RS, Brazil; ^4^Universidade Federal da Fronteira Sul (UFFS), Realeza-PR, Brazil; ^5^Universidade Federal de Goiás (UFG), Goiâna-GO, Brazil; ^6^Centro de Cirugía de Mínima Invasión Jesús Usón (CCMIJU), Cáceres-EX, Spain

## Abstract

*Introduction*. Gossypiboma is a rare surgical complication in small animals. The authors reported the laparoscopic diagnosis and treatment of an abdominal gossypiboma and chronic draining fistula postopen ovariohysterectomy (OVH) unresponsive to medical treatment in a bitch. *Case Presentation*. The patient had undergone OVH and exploratory laparotomy in other veterinary practice 3 years previously. The animal, presenting a chronic fistula, was then referred to UFSM Veterinary Hospital. Ultrasonographic examination revealed a structure compatible with a granuloma. For the laparoscopic procedure, a 3-port (two at 11 mm; one at 6 mm) access was used. Adhesiolysis and mass removal were performed by blunt dissection and bipolar electrocoagulation. The fistula was treated by mobilising the omentum through it. There were no intra- or postoperative complications. The chronic wound showed first intention healing. The mass was composed of fibrous tissues surrounding one surgical gauze. *Discussion and Conclusion*. The removal of a retained surgical gauze in the abdomen by laparoscopy has already been described in medicine. However, a laparoscopic approach for treating a fistulous draining tract due to a gossypiboma has not been previously reported in dogs. Laparoscopic exploration of the fistula allowed the use of a pedicled omental flap through infected sites to control chronic infection. Laparoscopic surgery can be used to identify and treat abdominal gossypiboma in dogs, including those with chronic abdominal sinus.

## 1. Introduction

The maintenance of gauze compresses inside the patient, also known as gossypiboma, textiloma, cottonoid, or muslinoma [1, 2], is a rare complication in small animals. Such foreign bodies are considered to cause infection and promote abscess formation and adhesion. Clinical signs can be absent for years [1], leading to fatal consequences in humans and animals [3, 4].

To the best of the authors' knowledge, this is the first case report of the use of laparoscopic procedure for the diagnosis and treatment, during the same surgical act, of a dog presenting a severe and chronically infected abdominal fistula due to gossypiboma. This study was reported according to the SCARE criteria [5].

## 2. Presentation of Case

A five-year-old female mongrel dog (10.8 kg) was referred from another Brazilian state to the Veterinary Hospital at the Federal University of Santa Maria (UFSM) for exploratory laparoscopy under diagnosis of an intra-abdominal mass with chronic fistulous draining tract that was unresponsive to a previous treatment.

The animal had undergone elective ovariohysterectomy at another veterinary hospital 3 years earlier. A few months later, the dog presented with a chron.ic draining fistula that was not responsive to clinical treatment with different classes of antibiotics, including cephalosporin, amoxicillin, clavulanic acid, and enrofloxacin. An exploratory laparotomy at another veterinary hospital revealed a mass next to the caudal pole of the right kidney, which was not removed or referred to biopsy for unknown reasons. Material was collected from the sinus for antibiogram analysis, showing sensitivity to amoxicillin-potassium clavulanate and cephalexin, among others, with the latter being the antibiotic prescribed and administered for approximately the next 4 months. However, the drainage did not cease. The animal was then referred to the UFSM for laparoscopic diagnosis and possible treatment.

In the clinical examination at the UFSM, the animal presented with a chronic fistula draining a purulent content located at the right flank close to the iliac crest. A drain, inadequate in size and type, was found inserted through the fistula. Ultrasonographic examination revealed a circumscribed structure caudal to the right kidney with acoustic shadowing resembling a granuloma. Elevation of plasma protein values (9.2 g/dL), thrombocytopenia (199 *μ*L), and mild leukopenia (5 *μ*L) due to lymphopenia (900 *μ*L) were observed in the total blood count. Serum biochemistry also revealed an increase in gamma-glutamyl transferase (21.64 UI/L). Then, the dog was referred to a minimally invasive surgery service of the hospital (SOMIV-UFSM, veterinary minimally invasive solutions) on the same day to investigate the intra-abdominal mass and the cause of the chronic infection. A prior computed tomography scan was not undertaken because it was unavailable and close to the region (in a radius of more than 300 km).

**Figure 1:**
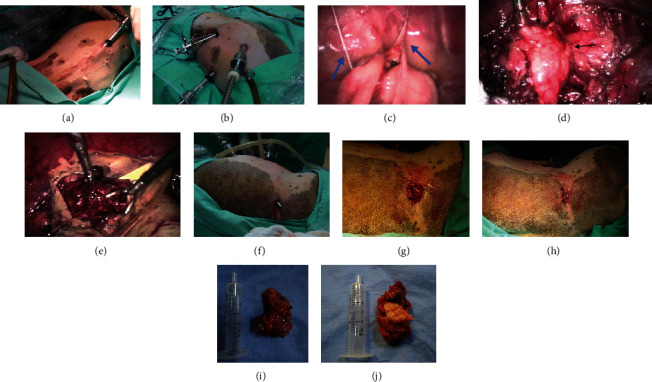
Laparoscopic diagnosis and treatment of gossypiboma in a female dog. (a) Placement of the first port (11 mm) in the ventral midline. (b) After lateralisation of the patient, two other trocars (6 mm and 11 mm) were positioned laterally to the first one in triangulation. (c) Laparoscopic aspect of the gossypiboma is envolved by the omentum; blue arrows indicate fibrous adhesions with the abdominal wall. (d) Gossypiboma (black arrows) after adhesiolysis; K: kidney. (e) Introducing gossypiboma into the tissue removal bag. (f) Cannula passage through the fistula after its hydrodynamic debridement. (g) Aspect of the chronic wound associated to the fistula after surgical debridement and laparoscopic positioning of the omentum through the muscular wall. (h) Aspect of the sutured wound. (i, j) Macroscopic aspect of gossypiboma before and after opening the fibrous tissue surrounding the surgical gauze.

After medication with ketamine (10 mg/kg), midazolam (0.4 mg/kg), and morphine (0.4 mg/kg), adequate hygiene and hair clipping of the flank were provided. Anaesthesia was induced with propofol and maintained with isoflurane in 100% oxygen. The dog was positioned in dorsal recumbency for laparoscopic surgery. The sinuous path was irrigated after drain removal. Then, a 3-port laparoscopic approach was selected. After skin antisepsis, an 11 mm port was placed on the preumbilical midline using the open technique (Hasson-modified) (Figure 1(a)). The CO_2_ pneumoperitoneum was set to 12 mmHg. Positioning was changed to left lateral recumbency, and another 11 mm port was established craniolateral to the first one. Finally, a third 6 mm port was placed caudolaterally in triangulation (Figure 1(b)).

The first intra-abdominal inspection revealed a mass at the right kidney pole, at the right ovary site, as well as intraperitoneal adhesions (Figure 1(c)). The mass was attached to the omentum, inner abdominal wall, and small bowel. In addition, there were omentum adhesions at the midline. Adhesiolysis, prophylactic haemostasis, and peritumoral dissection were performed using a laparoscopic bipolar electrosurgical device (Lina Tripol PowerBlade, LINA Medical Inc., Denmark) distributed by WEM Electronic Equipment Ltd., Ribeirão Preto, SP, Brazil) and Kelly forceps (Karl Storz, Tuttlingen, Germany), preserving the right ureter, which coursed near the mass. Short gauze threads were identified, while thick fibrous attachments to the right inner flank wall were transected. The specimen was withdrawn using a retrieval bag (LapSac, Cook, Bloomington, USA) following an 11 mm working port small enlargement (approximately 1 cm) (Figures 1(d) and 1(e)).

The tip of a 5 mm cannula attached to a vacuum cleaner was inserted through the fistula, which was thoroughly rinsed with 0.1% povidone-iodine solution (Figure 1(f)). A cutaneous incision around the fistula ostium and debridement of the granulation tissue external to the cavity was performed. Keeping the pneumoperitoneum, an omentum segment was apprehended and positioned through the sinus tract, covering the debrided tissue (Figure 1(g)). Omentopexy was performed using 2-0 USP polyglactin 910, and skin suture over the omentum was performed with interrupted horizontal mattress sutures using 3-0 USP nylon (Figure 1(h)).

No intra- or postoperative complications were observed. The total surgical time, from first incision to skin closure, was approximately 200 min. The animal recovered uneventfully in the early postoperative period and was discharged the same day with cephalexin, tramadol, and meloxicam (pharmacological doses). All wounds had first intention healing, and all sutures were removed 8 days after surgery.

Postsurgical immediate macroscopic observation of the specimen revealed a surgical gauze involving a thick fibrous capsule (Figures 1(i) and 1(j)). Histological assessment of the capsule identified blood vessels, collagen fibres, and inflammatory infiltrate with epithelioid macrophages, giant polynucleate cells, lymphocytes, and plasmocytes. These findings confirmed the diagnosis of gossypiboma.

The patient recovered completely, and the animal did not present any complications for 5 years. In contact via telephone, the owner reported that the animal was diagnosed with a cutaneous hemangiosarcoma and died due to complications related to its management at another veterinary facility.

## 3. Discussion

The present report highlights the use of laparoscopy to assess and treat a complicated case of gossypiboma with a fistulous draining tract in a dog. This approach has not been previously reported in the literature. Moreover, this case report describes the innovative use of a laparoscopic-assisted omental flap through the fistula to treat a chronically infected wound.

The use of laparoscopy in the removal of abdominal retained surgical gauze has been described in humans [2, 6] and in the removal of migratory foreign bodies in cheetahs (*A. jubatus*) [7]. Similar to the present report, Hartmann et al. [7] reported that haemostasis was achieved using bipolar electrosurgery to remove the granulomatous tissue surrounding the foreign body. The use of standard bipolar electrosurgery in these situations should be extremely judicious due to the risk of causing thermal lesions to important organs that surround or may be attached to the mass, as we observed in relation to the ureter and duodenum that are closely related to the gossypiboma. In this case report, meticulous tissue handling and cautious use of bipolar diathermy provided optimal outcomes, demonstrating that electrosurgery is a good alternative for similar conditions.

We chose to insert the first portal in the midline to evaluate the abdominal cavity as far as possible and to choose the best location for the other two portals. After assessing the cavity, the second and third portals were placed on the right flank, in a position similar to that used for laparoscopic nephrectomy [8]. Adhesions in the midline due to previous celiotomies were expected, and they were noticed when the cavity was evaluated by laparoscopy. We used the open portal placement technique, because it is considered safe for patients with previous abdominal surgery. Using this approach, it is possible to proceed with adhesiolysis through the incision before placing the first portal.

In the current report, it was not possible to establish a safe dissection plane at the site of the gossypiboma attachment to the inner abdominal wall, close to the fistulous tract. Therefore, laparoscopic hydrodynamic debridement was selected to explore the fistula and rinse the saline solution from the abdominal fistula site to the cutaneous ostium. This provided fluid drainage from the inside, avoiding intraperitoneal contamination. Subsequently, the sinus tract and the defect were covered with an omentum flap, which was based on previous successful outcomes using a pedicled omental flap through infected sites [9].

According to a previous study, the use of an omental flap can control chronic infections [10]. Omentum is widely known to promote neovasculogenesis; improve lymphatic drainage; provide lymphocytes, stem cells, complement proteins, and immunoglobulins; and enhance the release of growth factors and energy supply. It also avoids the formation of undesirable intraperitoneal adhesion [9].

Washing wounds and cavities using 0.1% povidone-iodine is unusual in small animals. However, this is a practice described in paediatric surgery in medicine [11], which we eventually used in a few cases under special conditions of abdominal cavity contamination. Considering the severity of the infection in this specific patient, we chose to perform exhaustive washing of the sinuous path with 0.1% povidone-iodine. There were no signs indicating peritonitis or cellulitis caused by the washing solution.

Considering the severity and chronicity of the fistula, laparoscopic-assisted thorough irrigation and omentalisation was feasible and provided optimal management of the draining tract. Moreover, the authors suggest that such a minimally invasive approach should be attempted before performing exploratory celiotomy to manage intraperitoneal fistulas in dogs.

## 4. Conclusion

The laparoscopic approach provides accurate identification and treatment of abdominal gossypiboma in dogs, including complex cases of complicated chronic abdominal fistulas.
